# Genomic Analysis of SARS-CoV-2 Alpha, Beta and Delta Variants of Concern Uncovers Signatures of Neutral and Non-Neutral Evolution

**DOI:** 10.3390/v14112375

**Published:** 2022-10-27

**Authors:** Monika Klara Kurpas, Roman Jaksik, Pawel Kuś, Marek Kimmel

**Affiliations:** 1 Faculty of Automatic Control, Electronics and Computer Science, Silesian University of Technology, Akademicka 16, 44-100 Gliwice, Poland; monika.kurpas@polsl.pl (M.K.K.); roman.jaksik@polsl.pl (R.J.); pawel.kus@polsl.pl (P.K.); 2 Department of Statistics and Bioengineering, Rice University, 6100 Main Street, Houston, TX 77005, USA

**Keywords:** coronavirus, SARS-CoV-2, variant of concern, mutation, site frequency spectrum

## Abstract

Due to the emergence of new variants of the SARS-CoV-2 coronavirus, the question of how the viral genomes evolved, leading to the formation of highly infectious strains, becomes particularly important. Three major emergent strains, Alpha, Beta and Delta, characterized by a significant number of missense mutations, provide a natural test field. We accumulated and aligned 4.7 million SARS-CoV-2 genomes from the GISAID database and carried out a comprehensive set of analyses. This collection covers the period until the end of October 2021, i.e., the beginnings of the Omicron variant. First, we explored combinatorial complexity of the genomic variants emerging and their timing, indicating very strong, albeit hidden, selection forces. Our analyses show that the mutations that define variants of concern did not arise gradually but rather co-evolved rapidly, leading to the emergence of the full variant strain. To explore in more detail the evolutionary forces at work, we developed time trajectories of mutations at all 29,903 sites of the SARS-CoV-2 genome, week by week, and stratified them into trends related to (i) point substitutions, (ii) deletions and (iii) non-sequenceable regions. We focused on classifying the genetic forces active at different ranges of the mutational spectrum. We observed the agreement of the lowest-frequency mutation spectrum with the Griffiths–Tavaré theory, under the Infinite Sites Model and neutrality. If we widen the frequency range, we observe the site frequency spectra much more consistently with the Tung–Durrett model assuming clone competition and selection. The coefficients of the fitting model indicate the possibility of selection acting to promote gradual growth slowdown, as observed in the history of the variants of concern. These results add up to a model of genomic evolution, which partly fits into the classical drift barrier ideas. Certain observations, such as mutation “bands” persistent over the epidemic history, suggest contribution of genetic forces different from mutation, drift and selection, including recombination or other genome transformations. In addition, we show that a “toy” mathematical model can qualitatively reproduce how new variants (clones) stem from rare advantageous driver mutations, and then acquire neutral or disadvantageous passenger mutations which gradually reduce their fitness so they can be then outcompeted by new variants due to other driver mutations.

## 1. Introduction and Background

In this study, we concern ourselves with the week-by-week chronology of evolution of the Severe Acute Respiratory Syndrome Coronavirus 2 (SARS-CoV-2) genomes as an illustration of emergence of variants of concern (VOC) of the virus and other elements of virus evolution. For this purpose, we downloaded almost 5 million genomic sequences from the GISAID database, collected from week 1 until week 97 of the pandemic. Using the original Wuhan consensus genome as a reference, we aligned all the sequences and split these into subsets, each including the sequences registered in a 1-week-long window. In each of the 97 time points, we created a list of variant sites at which the genomes differed from the Wuhan genome sequence, be it nucleotide substitutions, deleted nucleotides or non-sequenceable sites or site runs.

We categorized the genomes into disjoint subsets: non-variant of concern (non-VOC) mostly present in the early period of the pandemic, the Alpha (“British”) VOC, the Beta (“South African”) VOC and the Delta (“Indian”) VOC. In our data series, we observe the early stages of the Omicron VOC but not the latter’s divergence into substrains.

We decided not to include Omicron variant data in our analysis. One reason is the staggering count of genomes and the very rich diversification of Omicron variants. Therefore, we focused on the relatively simple “traveling wave” pattern of the pre-Omicron period. Simulations in the end section of Results qualitatively reproduce the pattern of the pre-Omicron era but would not be helpful to understand the Omicron data.

In our considerations, the benchmark is the hypothesis of the strongly asexual evolution of the virus, which implies that all VOC are clonal and share the same ancestral sequence. Recombination or repeated instances of variant emergence may contradict this hypothesis in its simple form. Recombination may occur, for example, if a mixture of more than 1 strain infects a host cell where they may trade portions of their genomes.

The SARS-CoV-2, which caused the current COVID-19 pandemic is a single-stranded RNA virus and it is expected to mutate at a pace of $10^{-4}$ nucleotide substitutions per site per year [1,2]. Although most of these mutations are either deleterious or neutral, some of them may impact the transmissibility and infectivity of the emerging strain. In addition, the accumulation of mutations may lead to immune escape, leading to an increased likelihood of reinfection. These features are observed in some of the VOC [3,4]. We turn now to some background information. It has to be noted that many recent papers discuss adaptation and purifying selection to variants evolution. A helpful introduction and recent literature review are provided by Neher [5], and we return to the subject in the Discussion.

### 1.1. B.1.1.7 (Alpha) Variant

The B.1.1.7 variant, later recognized as a variant of concern, was first detected in November 2020 in a sample taken on 20 September 2020 in the United Kingdom. On 14 December 2020, Public Health authorities in England reported a new SARS-CoV-2 variant referred to as Variant Under Investigation (later recognized as VOC) [6]. The B.1.1.7 variant is characterized by 15 non-synonymous mutations and 3 deletions [7,8] (Table A1). Several amino acid mutations are observed in the S protein of the Alpha variant, including D614G, N501Y and deletions H69-V70. It was reported that the S-receptor-binding domain (RBD) N501Y mutation increases the binding affinity to the ACE-2 receptor, facilitating transmission [9]. With transmissibility increased by 43–90% [10,11], and about a twofold replicative advantage [12], the Alpha variant began to spread, quickly outnumbering the original Wuhan strain.

### 1.2. B.1.351 (Beta) Variant

Another of several SARS-CoV-2 variants believed to be of particular importance was announced for the first time on 18 December 2020 by South Africa’s health department. The first sample was detected in the Nelson Mandela Bay metropolitan area of the Eastern Cape province of South Africa in October 2020. The B.1.351 variant is characterized by 17 mutations, with 9 of them in the Spike protein coding region [13] (Table A2), including three critical mutations in the RBD (K417N, E484K and N501Y) that impact viral fitness, transmissibility and survival adaptations [9].

### 1.3. B.1.617.2 (Delta) Variant

The B.1.617.2 variant appeared in Maharashtra state in India in October 2020 [14,15] and quickly became dominant in most countries. This variant is characterized by rapid transmission and spread, which is indicative of selective advantages against other VOC such as B.1.1.7 or B.1.351. Studies suggest a high risk of hospitalization compared with the Wuhan strain or the B.1.1.7 variant [16,17] and higher potential of immune evasion [15,18,19]. The B.1.617.2 variant is characterized by 2 deletions and 18 mutations, with 9 of them in the Spike protein coding region [19] (Table A3). Some of the most important Delta variant mutations are the P681R mutation present in the Spike insertion region, which distinguishes SARS-CoV-2 from, among others, bat coronaviruses [20] and T478K. Spike mutation, which has impact on infectivity and pathogenesis, facilitates viral replication and is potentially responsible for antibody escape [19,21].

We are exploring the history of each of the segregating sites present in Alpha, Beta and Delta VOC. We are trying to answer the question of whether defining mutations were accumulating gradually until they formed a sequence characteristic of the Alpha, Beta and Delta variants, or whether this phenomena can be explained by the recombination of two genomes with subsets of mutations.

We then use the longitudinal data of evolution of mutation frequencies to classify the genetic forces active at different ranges of the mutational spectrum. We investigate neutrality of the mutations at the lowest frequencies with the Griffiths–Tavaré theory [22]. At the mid-frequency range, we look for negative selection using the Tung–Durrett model [23] assuming clone competition. These results add up to a model of genomic evolution. Certain observations, such as mutation “bands” persistent over the epidemic history, suggest contribution of genetic forces different from mutation, drift and selection, including recombination or other genome transformations. In addition, we investigate a“toy” mathematical model based on the Tug-of-War concept [24] to verify if it may qualitatively reproduce how new variants (clones) stem from rare advantageous driver mutations, and then acquire neutral or disadvantageous passenger mutations which gradually reduce their fitness.

## 2. Materials and Methods

### 2.1. Multiple Sequence Alignment and Sample Preparation

The analysis was carried out using 4,276,493 nucleotide sequences of SARS-CoV-2 genomes, after filtering (rejection of those with incomplete collection date) 4.7 million sequences downloaded from the GISAID (Global Initiative on Sharing Avian Influenza Data) database [25,26]. Samples were dated from 24 December 2019 to 27 October 2021. The list of accession numbers for several important sequences (first sequence collected in Wuhan, sequences of first official cases of Alpha and Beta variant and one of the characteristic Delta variant sequences) can be found in Appendix B.

The sequences were aligned using Nextclade sequence aligner [27], with NC_045512.2, the first sequenced SARS-CoV-2 genome from Wuhan [28], as a reference sequence to accelerate the calculations and to identify gene positions inside the Multiple Sequence Alignment (MSA) created. To align such a high number of whole viral genome sequences efficiently, we had to disregard insertions. One reason for this is that, as we observed using much smaller samples of the order of $10^{5}$ genomes, alignments involving insertions are significantly longer and reach up to twice the length of the accepted genome length. Moreover, combining insertions and deletions leads to a very slow progress in constructing alignments, with no assurance of concluding it in a realistic time. We acknowledge that this leaves out potentially important markers meriting monitoring, such as those listed in Garushyants et al. [29].

### 2.2. Algorithms to Generate Weekly Statistics of Viral Genomes

**Figure A1:**
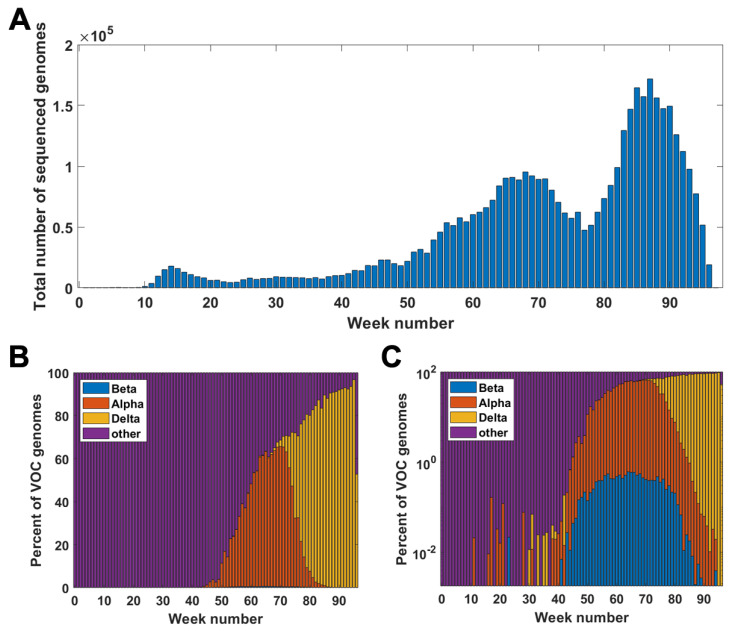
Genomes in the GISAID database week by week. (**A**) Total number of genomes sequenced in particular weeks of the pandemic. (**B**,**C**) Percentage of VOC genomes among all sequenced genomes in given weeks of the pandemic; linear scale (**A**); log scale (**B**).

We created statistics for each week since the beginning of the pandemic by recording the total number of genomes as well as the number of Alpha, Beta and Delta variant genomes recorded in a given week (see Figure A1).

### 2.3. Studies of Segregating Sites

Segregating sites characteristic of Alpha, Beta and Delta SARS-CoV-2 variants (see Section 1 and Table A1, Table A2 and Table A3) were identified from the alignment based on comparison with reference sequence. The length of the Alpha variant segregating sites’ subsequence is 36 nucleotides, in the case of the Beta variant segregating sites, the subsequence has 33 nucleotides, and for Delta, the segregating sites’ sequence has 33 nucleotides. In all cases, the segregating sites’ vectors include positions of deletions, which are aggregated to one segregating site.

We reviewed all 4,276,493 subsequences of SARS-CoV-2 genomes. For each position in the subsequence, we checked whether a given genome has VOC-defining mutations in corresponding places. Then, if this was the case, we saved the accession number and collection date of such genomes. Having these data enabled us to quantify the change in the abundance of individual mutations over time and to study possible subsets of 2, 3, 4 and so forth mutations present together in one genome, as well as to determine the dates when such subsets arose. We compared observed counts of mutation subsets in tested samples to the number of subsets expected under equally likely random assortment, given the total count of segregating sites, calculating the binomial coefficient

nk=n!k!(n−k)!

where *n* is total number of segregating sites and *k* is the number of segregating sites in a given subset.

### 2.4. Studies of the Site Frequency Spectra

#### 2.4.1. Definition of the Site Frequency Spectrum (SFS)

**Figure 1:**
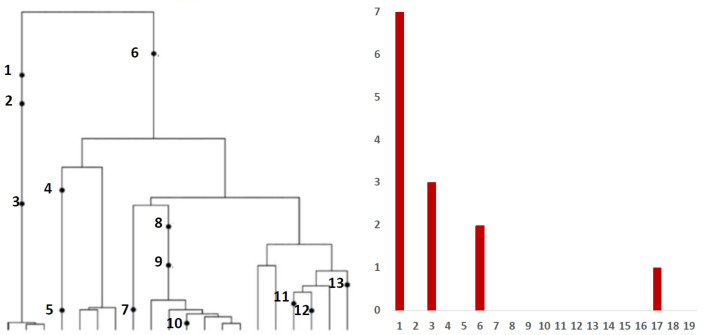
**Left** panel: genealogy of a sample of n=20 genomes including 13 mutational events, denoted by black dots. Mutations 4, 5, 7, 10, 11, 12 and 13 (total of 7 mutations) are present in a single genome, mutations 1, 2 and 3 (total of 3 mutations) are present in three genomes, mutations 8 and 9 (2 mutations) are present in six genomes and mutation 6 (1 mutation) is present in 17 genomes. **Right** panel: the observed site frequency spectrum (SFS), $S_{20}\left(1\right)=7$ , $S_{20}\left(3\right)=3$ , $S_{20}\left(6\right)=2$ and $S_{20}\left(17\right)=1$ , other $S_{n}\left(k\right)$ equal to 0.

Inference from evolutionary models of DNA often exploits summary statistics of sequence data, a common one being the so-called Site Frequency Spectrum (SFS). In a sequencing experiment with a known number of sequences, we can estimate for each site, at which a novel somatic mutation has arisen, the number of genomes that carry that mutation. These numbers are then grouped into sites that have the same number of copies of a mutant. Figure 1 (based on [30]; modified) gives an example with time running down the page. The genealogy of a sample of n=20 genomes includes 13 mutational events. We can see that mutations 4, 5, 7, 10, 11, 12 and 13 (a total of 7 mutations) are present in a single genome, mutations 1, 2 and 3 (total of 3 mutations) are present in 3 genomes, mutations 8 and 9 (a total of 2 mutations) are present in six genomes and mutation 6 is present in 17 genomes. If we denote the number of mutations present in *k* genomes by $S_{n}\left(k\right)$ , we see that in this example, $S_{n}\left(1\right)=7$ , $S_{n}\left(3\right)=3$ , $S_{n}\left(6\right)=2$ and $S_{n}\left(17\right)=1$ , with all other $S_{n}\left(j\right)$ equal to 0. The vector $\left(S_{n}\left(1\right),S_{n}\left(2\right),\ldots ,S_{n}\left(n-1\right)\right)$ is called the (observed) Site Frequency Spectrum, abbreviated to SFS. It is a convention to include only sites that are segregating in the sample, that is, those for which the mutant type and the ancestral type are both present in the sample at that site. Mutations that occur prior to the most recent common ancestor of the sampled genomes will be present in all genomes in the sample; these are not segregating and are called truncal mutations.

#### 2.4.2. SFS under Infinite Sites Model and Exponential Growth

The hypothesis of selective neutrality, leading to the “neutral” theory of evolution, is credited to Kimura [31]. The theory assumes that the number of mutations that have occurred by random stochastic processes without selective impact strongly exceeds the number of mutations affected by selection.

Under neutrality, in the framework of the infinite sites model (ISM), Griffiths and Tavaré [22] provide a general coalescent framework. For the expected number $ES_{n}\left(k\right)$ of mutant sites having *k* copies of the mutant in a sample of size *n*, drawn from a Wright–Fisher population model with size changing deterministically in the past, under the ISM, they showed among others that

(1) $$ES_{n}\left(k\right)=\theta \sum_{j=2}^{n-k+1}jp_{nj}\left(k\right)\;ET_{j},$$

where

pnj(k)=n−k−1j−2/n−1j−1,

where $T_{j}$ denotes the coalescence times for the model with arbitrary functional form of growth or decline of the population in the past. The expectations are generally difficult to derive analytically, and therefore it is convenient to consider the approximations provided by Durrett [32], who showed that if the population has been growing exponentially with growth rate *r*, i.e., $N\left(t\right)=Ne^{rt},\;t<0$ , where *N* is the present population size, then as N→∞ ,

(2) $$ES_{n}\left(k\right)\to \frac{\theta }{r}\frac{n}{k(k-1)},\;k=2,\ldots ,n-1,$$

while

(3) $$ES_{n}\left(1\right)∼\frac{\theta n\ln(rN)}{r},$$

where ∼ denotes asymptotic equivalence. This latter term follows directly from one of the versions of Griffiths and Tavaré [22] results. A slightly more accurate approximation by Durrett [32] for a finite *N* has the form

(4) $$ES_{n}\left(1\right)\approx \frac{\theta n}{r}\sum_{1\;\leq k\;\leq Nr}\frac{k}{\left(n+k)(n+k-1\right)}.$$

Relevance of the singletons for DNA sequencing data is questioned by many, since low-frequency variants are routinely pruned by data-cleaning algorithms to avoid confusion with sequencing errors. Concerning non-singletons, i.e., doublets, triplets and so forth, expression (2) implies that the total count of these mutations is equal to

(5) $$A\;=\;\sum_{k=2}^{n-1}\;ES_{n}\left(k\right)\;\approx \;\sum_{k=2}^{n-1}\;\frac{\theta }{r}\frac{n}{k(k-1)}\;=\;n\frac{\theta }{r}\left(1-\frac{1}{n-1}\right)\;=\;\frac{\theta }{r}\frac{n(n-2)}{n-1}$$

Operationally, expressions (2) and (3) are the simplest to use. Arguably, virus evolution might be better described by the linear birth–death processes and not Wright–Fisher or Moran model with exponential growth; we should in principle use the corresponding SFS expressions, such as those derived in Appendix E to Dinh et al. [30]. However, these latter involve Gauss hypergeometric functions and, numerically, they work very much like Griffiths–Tavaré expressions (see [30], Figure 3).

#### 2.4.3. SFS under Birth-and-Death Process Model with Mutation and Selection

**Figure 2:**
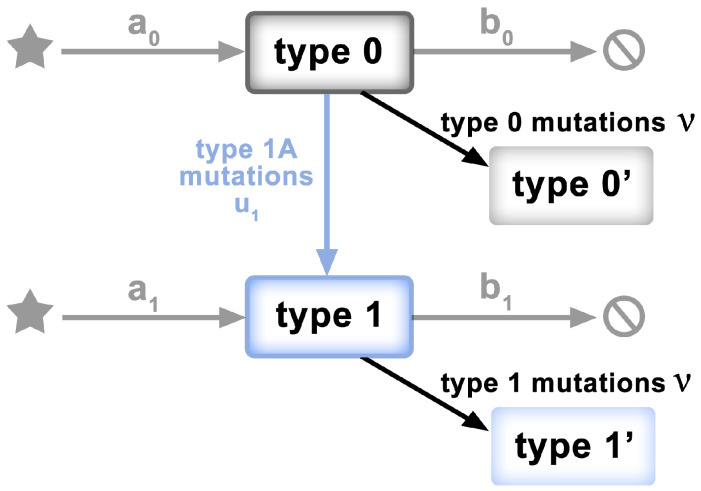
Schematic of the model of [23,33]. Clonal expansion begins with a single genome of the ancestral individual (Type 0). Type 0 individuals grow at rate $\lambda_{0}=a_{0}-b_{0}$ . Neutral mutations accumulate at rate ν . Type 0 individuals mutate to Type 1 at rate $u_{1}$ . Type 1 individuals grow at rate $\lambda_{1}=a_{1}-b_{1}$ , where $\lambda_{1}>\lambda_{0}$ .

To test the “mid-range” frequency mutations for departures from non-neutrality, we adopt a model of McDonald et al. [33], characterized mathematically by Tung and Durrett [23]. The model has the form of a two-type birth–death process, as depicted in Figure 2. Specifically,

- Clonal expansion begins with a single genome of the ancestral individual (viral genome)—type 0.
- Type 0 individuals give birth at rate $a_{0}$ and die at rate $b_{0}$ , so the exponential growth rate is $\lambda_{0}=a_{0}-b_{0}$ .
- Neutral mutations accumulate at rate ν during the individual’s life time; not only at birth.
- Type 0 individuals mutate to type 1 at rate $u_{1}$ .
- Type 1 individuals give birth at rate $a_{1}$ and die at rate $b_{1}$ . Their exponential growth rate is $\lambda_{1}=a_{1}-b_{1}$ , where $\lambda_{1}>\lambda_{0}$ .
- Assumption: all type 1 mutants have the same growth rate

Under these hypotheses, if the fitnesses of the two types are $\lambda_{0}<\lambda_{1}$ , then the site frequency spectrum approximately follows the power curve, in our notation

(6) $$S\left(x\right)=Cx^{-(1+\alpha )},\;\;x=1,\ldots ,n$$

hence the tail $T\left(x\right)=\sum_{\xi >x}S\left(\xi \right)$ follows the law

(7) $$T\left(x\right)=\frac{C}{\alpha }\left(n^{-\alpha }-x^{-\alpha }\right),\;\;x=1,\ldots ,n$$

where $\alpha =\lambda_{0}/\lambda_{1}$ . This is due to the advantageous mutations that produce the founders of the type 1 population. As seen in the Results section, the mid-range frequency SARS-CoV-2 data conform to the power curve, though with α>1 , which corresponds to

$$\lambda_{0}>\lambda_{1}$$

i.e., to disadvantageous mutants. This latter assertion was not proved in [23]. However, similarly shaped tails T(x) are produced by the Tug-of-War model of selection, recently considered in [34]; see, e.g., Figures 5 and 13 there. Tug-of-War is a more complicated selection model, in which rare but strongly advantageous driver mutations compete with more frequent slightly disadvantageous passenger mutations [24].

### 2.5. Counting Genomes under Neutrality

In this case, the aim of neutrality testing is to determine whether the observed allele counts $a_{1},\ldots ,a_{n}$ conform to what is expected under null hypothesis assuming neutrality, given the sample size *n* and the observed number *k* of alleles in the sample. We use two types of models to investigate departures from neutrality. In both models, we assume that a new mutation is creating a new genome (new “allele”), i.e., we use the Infinite Allele Model (IAM). Under population size constancy, it is appropriate to use the Ewens Sampling Formula and its consequences. To allow for changing population size, we use the Griffiths–Pakes model [35] for the special case considered by Kimmel and Matthaes [36].

#### 2.5.1. Mutation–Drift Equilibrium under Constant Population in the IAM

The properties of a sample of *n* genes under infinitely many allele versions of the Wright–Fisher model are best summarized through the following (approximating) partition formula. Let us define $A=(A_{1},A_{2},\ldots ,A_{n})$ , where $A_{i}$ is the number of alleles present in exactly $a_{j}$ genomes (out of *n*) in the sample. With this definition, the following expression, known as Ewens Sampling Formula (ESF), was derived by Ewens [37] and Karlin and McGregor [38]:

(8) $$P\left(A=a\right)=\frac{n!\theta \sum a_{j}}{1^{a_{1}}2^{a_{2}}\ldots n^{a_{n}}a_{1}!a_{2}!\ldots a_{n}!S_{n}\left(\theta \right)},$$

where $a=(a_{1},a_{2},\ldots ,a_{n})$ and $S_{n}\left(\theta \right)$ are defined by

(9) $$S_{n}\left(\theta \right)=\theta \left(\theta +1\right)\left(\theta +2\right)\ldots \left(\theta +n-1\right)$$

where θ is the mutation rate (see next paragraph). Let us denote $\sum A_{j}$ , the (random) number of different allelic types seen in the sample, by *K*, and $\sum a_{j}$ , the corresponding observed number in a given sample, by *k*. We have $\sum jA_{j}=\sum ja_{j}=n$ . From Equation (8), the probability distribution of the random variable *K* can be obtained as

(10) $$P\left(K=k\right)=|S_{n}^{k}|\theta^{k}/S_{n}\left(\theta \right),$$

Quantity $S_{n}^{k}$ is the coefficient of $\theta^{k}$ in $S_{n}\left(\theta \right)$ and is called the Stirling number of the first kind. For testing purposes, we use the expression for the expectation of the sample frequency spectrum, conditional on K=k and given the sample size *n*.

(11) $$E\left(A_{j}|k,n\right)=\frac{n!}{j!(n-j)!}\frac{|S_{k-1}^{n-j}|}{|S_{k}^{n}|}$$

In this expression, the sequence of the $E(A_{j}|k,n)$ values for *j* = 1, 2, …, and *n* is the sample conditional mean frequency spectrum. The j=1 term is the singleton count, the j=2 term is the doublet count, and so forth.

#### 2.5.2. Mutation–Drift Equilibrium under Branching Process Population in the IAM

Griffiths and Pakes’ [35] process is a modification of the standard Bienayme–Galton–Watson branching process to allow individuals infinitely many possible identifiable types. In our application, the types are alleles (variants) of the SARS-CoV-2 genomic sequence identified by specific point mutations. From time t=0 , a non-mutant clone of genomes is evolving in discrete time according to a single-type branching process with probability μ per time step, a particle mutates and initiates a clone of a new previously non-existent type, which evolves according to the same rules as the original non-mutant clone. As a result, a set of clones of different types emerges, spawning further clones, some of which may die out. Kimmel and Matthaes [36] derived, using Griffith–Pakes’ theory, expected frequencies of allele classes such that an allele is in class *k* if it exists in *k* copies for a specific version of the process (further on).

The number of individuals at t=0 is defined as $Z_{0}=i$ . Let $G_{n}$ be the collection of individuals in generation *n* and let $Z_{n}$ denote their number. Each generation size depends on the previous generation size through the branching property

$$Z_{n\;+\;1}=\sum_{j\;=\;1}^{Z_{n}}\xi_{j,n},$$

where $\xi_{j,n}$ are independent identically distributed (iid) integer-valued random variables, which represent the number of offspring born to the jth member of $G_{n}$ . The distribution of $\xi_{j,n}$ is characterized by its probability generating function (pgf)

$$f\left(s\right)=\sum_{k\;=\;0}^{\infty }p_{k}s^{k},$$

where $p_{k}=P\left[\xi_{j,n}=k\right]$ , and it is assumed that $p_{0}+p_{1}<1$ , i.e., the branching process is non-trivial. We have $m=f^{\prime }\left(1\right)$ .

If an individual produces *j* offspring, then the number of progeny having the parental allele is distributed binomially with parameters *j* and 1−μ , hence its pgf is equal to ${\left(\mu +\left(1-\mu \right)s\right)}^{j}$ . This implies that any new allele is followed by a branching process of its like-type descendants with offspring pgf H(s)=f(μ+(1−μ)s) . This process is supercritical if its expected progeny count M=m(1−μ) is greater than 1. Let us denote $\Psi_{j}$ the long-term expected proportion of alleles with frequency j≥1 , which is the formula that we use to compute the theoretical distribution of Alu allele classes for given offspring pgfs. Asymptotically, at long times, these proportions tend to a limit.

***Linear fractional offspring distribution*** The process of creation of new viral genomes by mutation can be naturally described by the time-continuous age-dependent Markov branching process $\left\{Z_{t}\right\}$ (i.e., a process with exponentially distributed individuals’ lifelengths) with quadratic offspring pgf. If such a process is sampled at constant time intervals, the resulting discrete-time process $\left\{Z_{k\Delta t}\right\}$ is a Galton–Watson branching process with linear fractional pgf [39]. A unique property of the linear fractional case is that the iterations of the pgf can be computed explicitly and also are of linear fractional form. Let us start with the offspring pgf in the linear fractional case:

$$f\left(s\right)=1-\frac{b}{1-p}+\frac{bs}{1-ps}$$

As demonstrated by Kimmel and Mathaes [36], for the linear fractional case, we obtain the following computable expression

(12) $$\Psi_{j}=\left(1-s_{0}^{*}\right)\left\{\sum_{r\;=\;0}^{\infty }\frac{[{\left(m^{*}\right)}^{r}-1^{j-1}}{{\left[{\left(m^{*}\right)}^{r}-s_{0}^{*}\right]}^{j+1}}\right\}{\left\{\sum_{r\;=\;0}^{\infty }\frac{1}{{\left(m^{*}\right)}^{r}-s_{0}^{*}}\right\}}^{-1}$$

where $m^{*}=b^{*}/{\left(1-p^{*}\right)}^{2}$ is the overall expected growth rate of the process, and parameters $b^{*}=b\left(1-\mu \right)/{\left(1-p\mu \right)}^{2}$ and $p^{*}=p\left(1-\mu \right)/\left(1-p\mu \right)$ are subject to restrictions,

$$p^{*},b^{*}>0,\;\;b^{*}+p^{*}\leq 1.$$

To ensure that the process is supercritical, i.e., $m^{*}>1$ , an additional constraint is needed

$$p^{*}>1-\sqrt{b^{*}}.$$

### 2.6. Tug-of-War Model of Population Genetics in Moran Process Framework

We briefly describe the Tug-of-War model of McFarland et al. [24] in the Moran process version, referred to as Model B in Kurpas and Kimmel [34]. We consider a population of a fixed number *N* of haploid genomes of cells, viruses or other, each of them characterized by a pair of integers $\gamma_{i}=\left(\alpha_{i},\beta_{i}\right)$ corresponding to the numbers of driver and passenger mutations, respectively. The pair determines the fitness $f_{i}$ of the *i*-th genome by the equation

(13) $$\begin{array}{c} f_{i}=f_{i}\left(\alpha_{i},\beta_{i}\right)={\left(1+s\right)}^{\alpha_{i}}{\left(1-d\right)}^{\beta_{i}},\;\;i=1,\ldots ,N, \end{array}$$

where s>0 , the selective advantage of the driver, and d∈(0,1) , the selective disadvantage of the passenger, are parameters describing the selective advantage of driver mutations over passenger mutations; for rationale and further details, see [34]. There are two possible types of events: death replacement and mutation. Under the time-continuous Markov Chain model, the times to the nearest event are exponentially distributed. The parameter of the exponentially distributed time to the next death replacement event is equal to $\Sigma_{P}=\sum_{f_{i}\in P}f_{i}$ , where P is the set of fitnesses of genomes present before the death replacement event. We assume that the dying genome *i* is drawn from a uniform distribution on all the *N* genomes before death replacement. The replacing genome *j* is drawn from a distribution biased by fitness, with pmf $\left\{f_{j}/\Sigma_{P},f_{j}\in P\right\}$ . We allow the possibility that the replacing genome may be the same as the dying genome.

The parameter of the independently distributed exponential time to the next mutation is equal to N μ , where μ is the mutation rate per genome. The genome, chosen with probability 1/N , undergoes a mutation event, changing its state to either (α+1,β) or (α,β+1) with (conditional) probabilities p∈(0,1) and q=1−p , respectively. The time to the next event is random and exponentially distributed with parameter $\Sigma_{P}+N\mu$ , the total rate of death replacement and mutation events.

In the Results Section 3.5, we use this model as a “toy” to reproduce driver clone emergence observed in the data.

## 3. Results

### 3.1. VOC Timelines

**Figure 3:**
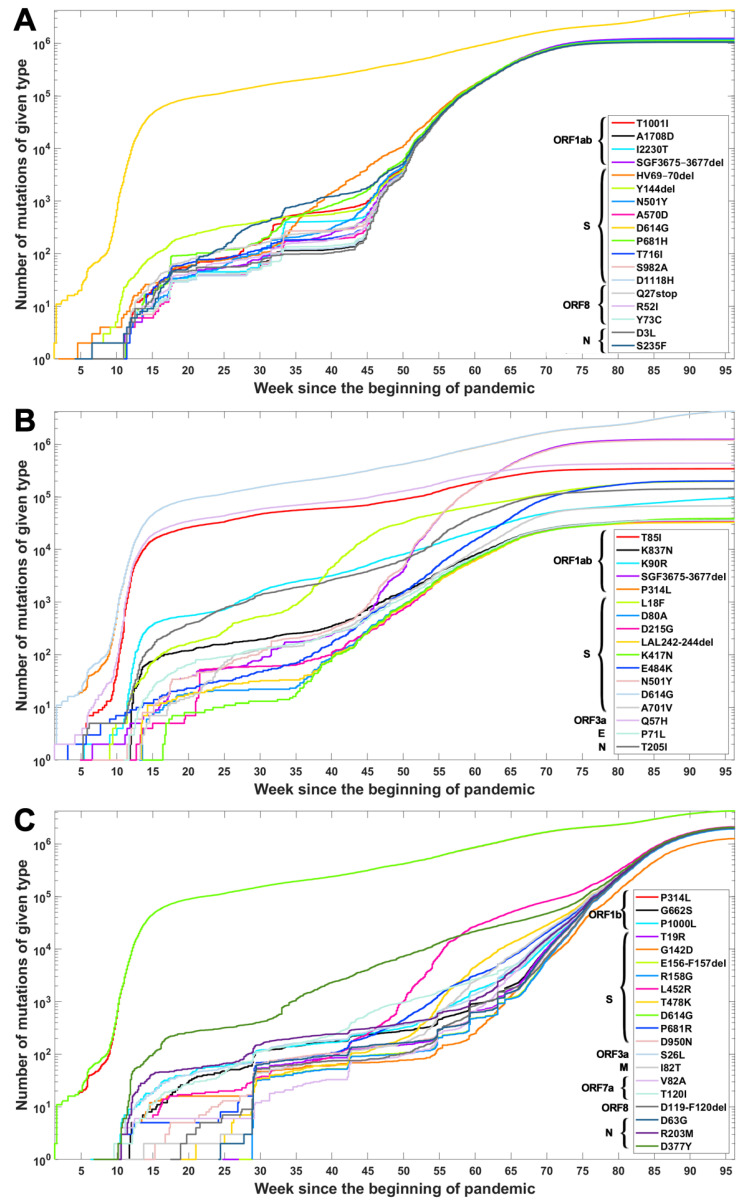
Cumulative count of genomes with variant of concern-defining (VOC-defining) mutations over time. Each curve depicts the number of genomes with given mutation that occurred up to a specified week. Mutations are listed in the same order as in Table A1, Table A2 and Table A3. (**A**) B.1.1.7 (Alpha) variant; (**B**) B.1.351 (Beta) variant; (**C**) B.1.617.2 (Delta) variant.

**Figure A2:**
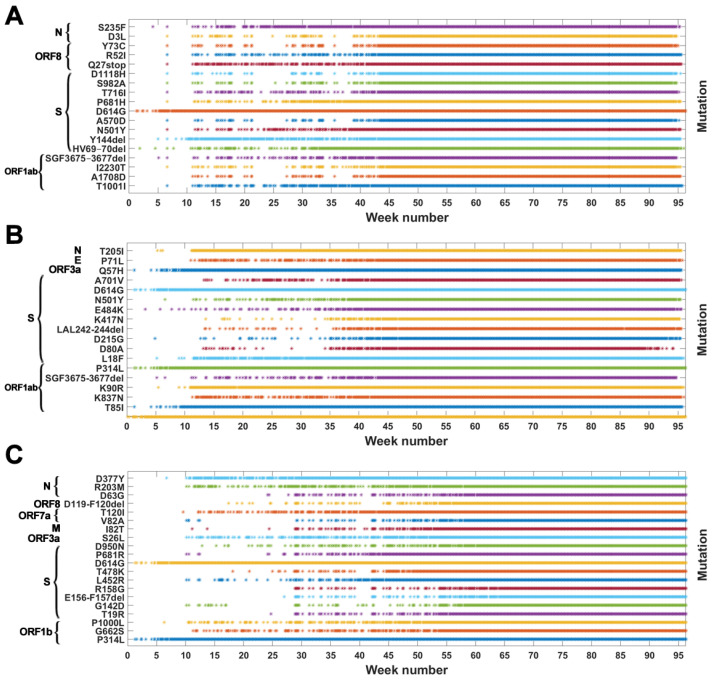
Genomes with VOC-defining mutations over time. Instances of appearance of genomes carrying given mutations at a given date are denoted by asterisks. Mutations presented are the same as those in Table A1, Table A2 and Table A3 (**A**) B.1.1.7 (Alpha) variant; (**B**) B.1.351 (Beta) variant; (**C**) B.1.617.2 (Delta) variant.

Based on the data from processing of subsequences containing segregating sites for Alpha, Beta and Delta SARS-CoV-2 variants, we generated timelines for each defining mutation. In Figure 3, we present cumulative plot showing changes in the dynamics of the increase in the count of individual VOC-defining mutations over time. In the Appendix B, a twin Figure A2 depicts discrete times at which the variant-defining mutations appeared. We observed that although genomes containing complete sets of VOC-defining mutations emerged in late 2020 (20 September 2020—the Alpha variant; 10 October 2020—the Beta variant; and October 2020—the Delta variant), specific mutations emerged as soon as the first weeks of the pandemic. This is especially true for such important mutations as D614G (Spike), P314L (ORF1b), Q57H (ORF3a) or T85I (ORF1a), classified as selectively advantageous [40,41,42,43]. Complete or near-complete sets of VOC-defining mutations emerged earlier in the case of the Alpha variant than in the remaining variants.

The dynamics of increase in the cumulative number of genomes with VOC-defining mutations is characterized by a number of growth spurts. This is likely caused by differences in the overall count of genomes sequenced on a given day (data are not normalized). However, for several mutations, we observed faster increases in count than in the remaining cases. Such surges in the number of sequenced genomes with given alteration can be explained by more frequent occurrences in populations or by uneven geographical distribution due to overrepresentation of data from Europe and the United States. Examples of such mutations (excluding D614G and P314L mentioned earlier) in the case of the Delta variant are D377Y (Nucleocapsid) and L452R (Spike; Figure 3C). For the Alpha variant, the alterations increasing faster in number are S235F (Nucleocapsid), HV 69–70 deletion (Spike) and Y144 deletion (Spike; Figure 3A). For the Beta variant, we observe the fastest increase in the number of genomes carrying the Q57H (ORF3a) and T85I (ORF1ab) mutations (at the beginning of the pandemic) and also N501Y (Spike), L18F (Spike) and SGF 3675–3677 (ORF1ab) deletion (Figure 3B).

### 3.2. Mutation Subsets and Their Frequency

**Figure 4:**
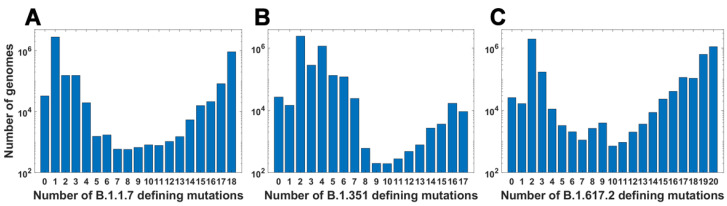
Frequency plots of genomes carrying a given number of VOC-defining mutations. (**A**) B.1.1.7 (Alpha) variant; (**B**) B.1.351 (Beta) variant; (**C**) B.1.617.2 (Delta) variant.

For all variants, we calculated how many genomes collected by 27 October 2021 carry a given number of mutations from the VOC-defining set (Figure 4A–C). In all variants, we observe that there is a large number of genomes carrying only one or two from the VOC-defining mutations but, especially in the case of the Alpha variant, there are also a lot of sequences carrying the complete set (927,610 genomes for the Alpha variant, 9144 genomes for Beta and 1,123,994 genomes for Delta) or an almost complete set. The least numerous are genomes having mutations in approximately one half of all segregating sites.

**Figure 5:**
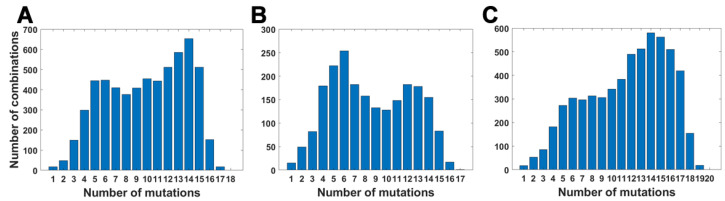
Number of unique subsets of VOC-defining mutations for a given mutation count. (**A**) B.1.1.7 (Alpha) variant; (**B**) B.1.351 (Beta) variant; (**C**) B.1.617.2 (Delta) variant.

We calculated the number of observed unique subsets of VOC-defining mutations for the Alpha (Table A4), Beta (Table A5) and Delta (Table A6) variants and compared them with the expected count of subsets under random assortment for given total mutation count. Results presented in Figure 5 and in Table A4, Table A5 and Table A6 demonstrate that the observed counts represent a minor fraction of the random assortments possible.

**Figure 6:**
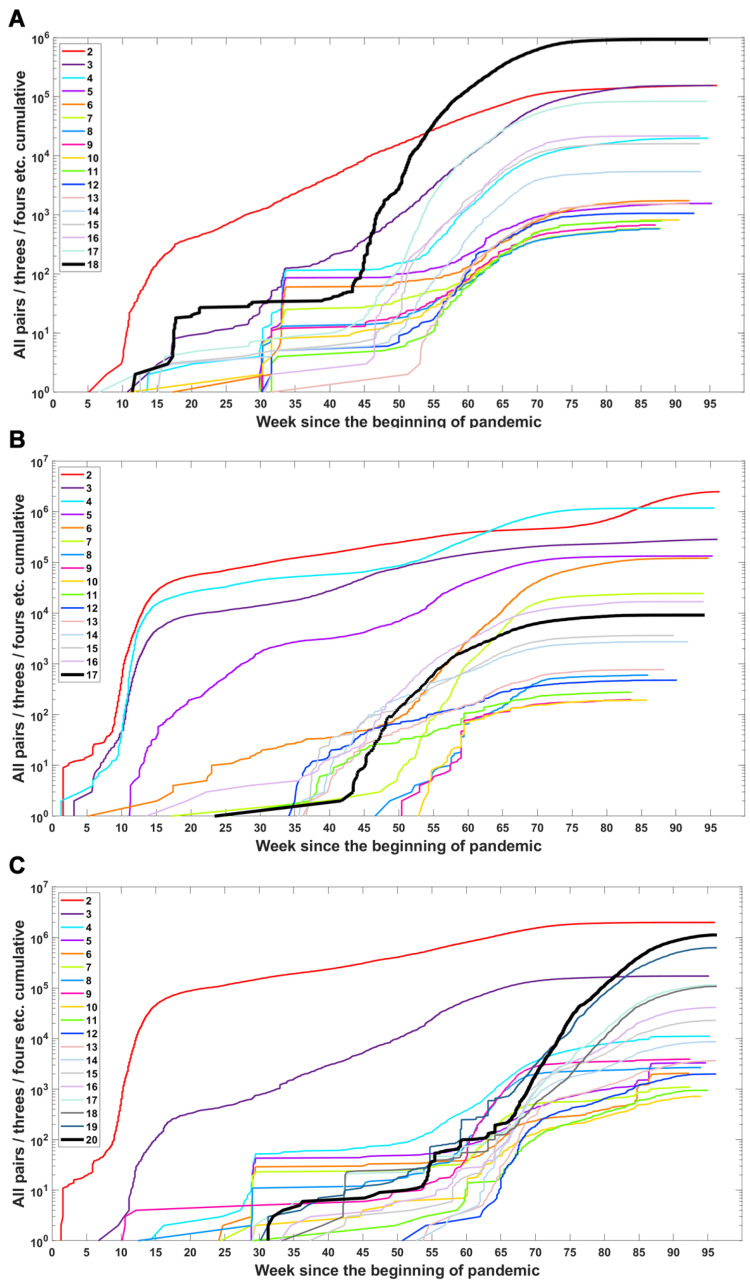
Accumulation of subsets of a given number of variant-defining mutations over time. Sets containing all VOC-defining mutations are marked by a thick black line. (**A**) B.1.1.7 (Alpha) variant; (**B**) B.1.351 (Beta) variant; (**C**) B.1.617.2 (Delta) variant.

**Figure A4:**
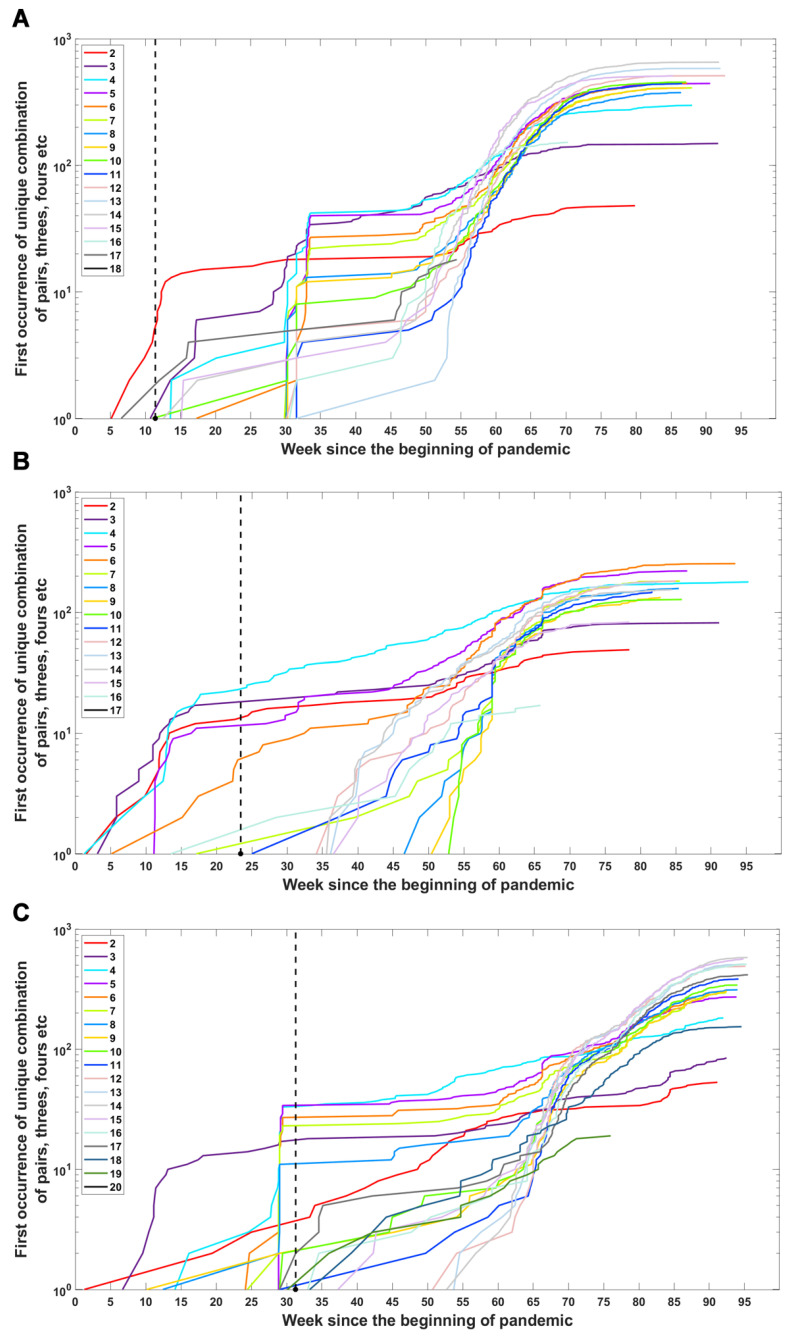
Cumulative count of unique subsets of a given number of mutations over time. (**A**) B.1.1.7 (Alpha) variant; (**B**) B.1.351 (Beta) variant; (**C**) B.1.617.2 (Delta) variant.

We investigated when subsets of a given number of mutations first emerged in time and what the dynamics of increase are in the number of total subsets (Figure 6) or unique subsets (Figure A4) over time. We observe that genomes carrying subsets of a higher number of mutations (even full set) emerge earlier than genomes carrying only part of them—in the case of the Delta VOC subsets containing 11, 12, 13, 14, 15 and 17, alteration appeared later than full or nearly full sets of variant-defining mutations (Figure 6C and Figure A4C). A similar pattern is also observed for the Alpha and Beta VOC (Figure 6A,B and Figure A4A,B).

### 3.3. Longitudinal Analysis of Mutations in the SARS-CoV-2 Genomes

In an attempt to understand the dynamics of the genomic evolution of the VOC of SARS-CoV-2, we carried out a longitudinal analysis of almost all mutations recorded in the GISAID genomes from week 1 through week 97 of the pandemic. The findings are described in the current section. Here, we are concerned with the “upper portion” of the mutation frequency range. This is why we ordered the almost 30,000 mutations at almost all genomic sites by the maximum relative frequency they have over the 97 week period we tackled. We chose 1000 sites ranking the highest with respect to this metric. In this way, we concentrate on sites at which the variants even transiently exceeded an admittedly neutral drift boundary and are likely to play a role in selection for or against the VOC. In the next sections, we focus on the lower and middle parts of the range.

The current section’s results are depicted in a series of figures in this section. We categorize the variants at any given site as:

- *substitutions*, whether to a specific nucleotide or to a different class of nucleotide such as, for example, purine to pyrimidine, pyrimidine to purine, and so forth;
- *deletions*, whether preserving (most of the times) or non-preserving the reading frame;
- *unknown or “N”*, in which the nucleotide was not determined in any way, but it was not deleted.

Sites which do not belong to any of the three categories are not counted. The three-way categorization is carried out for all genomes ascertained during a given week, so we obtain a series of snapshots of frequencies of mutations at all sites at which such mutations were recorded. These are not “trajectories” in the real sense, however, they allow tracking evolution of the viral genomes. Inclusion of the “N” category leads to puzzling results. Therefore, for now, we limit ourselves to substitutions and deletions and return to the “N”’s later on.

**Figure 7:**
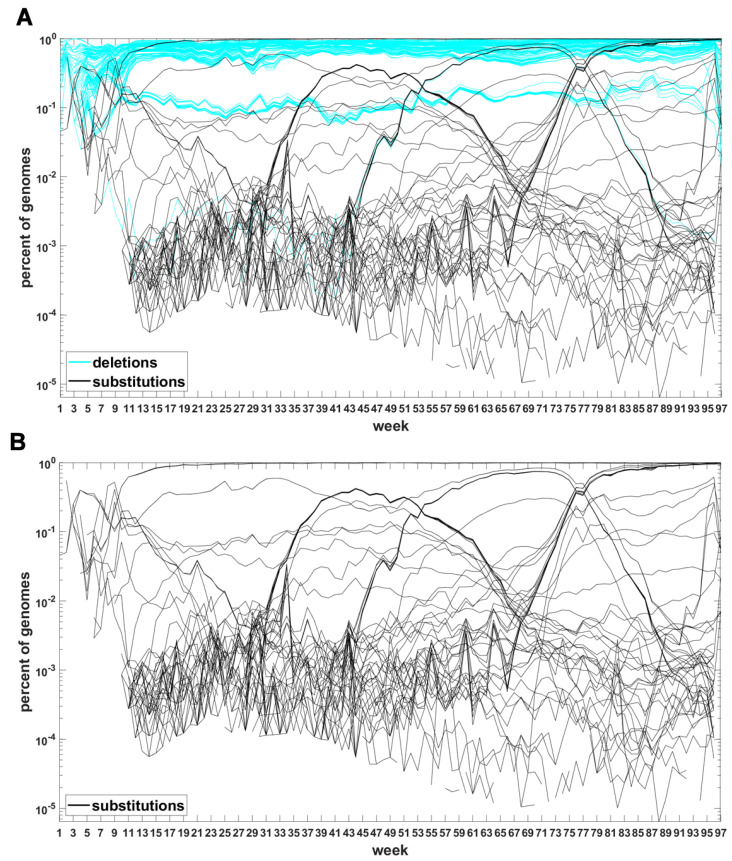
Time trajectories of frequencies of the alterations of specified type among top 1000 mutations observed in all genomes. (**A**) substitutions (black) and deletions (blue); (**B**) substitutions. Variant-defining sites excluded.

The classification seems to lead to interesting results. Figure 7 depicts time trajectories of frequencies of the top 1000 mutation sites of different categories, scaled to the total count of genomes recorded in a given week, in semi-logarithmic scale. Black curves mark nucleotide substitutions, while blue curves mark deletions. VOC-defining mutations (listed in Table A1, Table A2 and Table A3) are excluded in these, but not all, graphs. Let us notice that despite the exclusion, we observe the ∩-shaped bands that track the VOC. These are mutations, many of them synonymous, that “accompany” VOC, although they are not included in the standard VOC-defining sets. The one associated with the Alpha VOC peaks around week 70, while the one associated with the Delta VOC around week 95. The ∩-shaped band peaking around week 40 belongs to the B.1.177 lineage (also known as the “Spanish variant”), though not to any majorly recognized VOC. The band corresponding to the Beta VOC cannot be noticed because of the low frequency of these VOC genomes.

**Figure A5:**
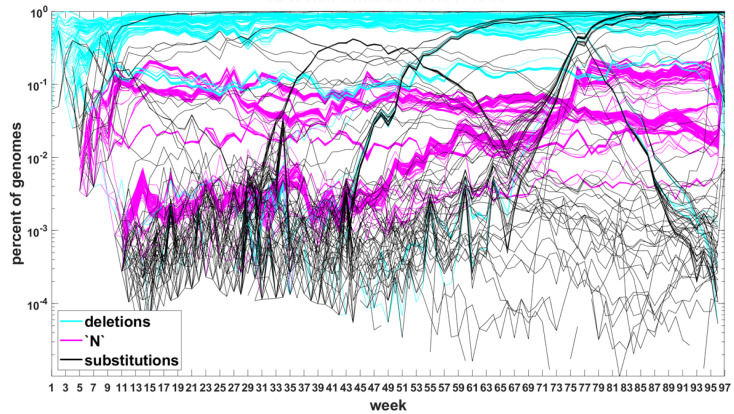
Time trajectories of frequencies of the top 1000 mutation sites of different categories: substitutions (black), deletions (blue) and unknown (magenta) observed in all genomes.

Figure A5 depicts the time trajectories of frequencies of the top 1000 mutation sites of different categories, including the non-identifiable variants (“N”-s; marked by magenta curves). These latter tend to be grouped along regions of the genomic sequences. Routine analysis of predicted RNA secondary structures at these stretches of “N”-s (not shown) does not seem to indicate an obvious imbalance of the stem-to-loop ratio or other features detectable by visual inspection. Nevertheless, their evolutionary importance seems doubtful, since they may coincide with regions difficult to sequence. Another observation concerns bands of mutations running horizontally through all VOC history, such as several black and blue lines at frequency ca. $10^{-1}$ in Figure 7A,B. If they correspond to “real” mutation sites, they are difficult to reconcile the clonal origin of the VOC. An apparent explanation is recurring substitution or deletion at some region, or frequent recombination with a persistent type that has the deletion and is not VOC-specific. This does not concern the thick blue band in Figure A5 at frequencies close to 1.0 , which likely belongs to deletions that occurred after the Wuhan ancestral genome and before the subsequent variant genomes.

**Figure 8:**
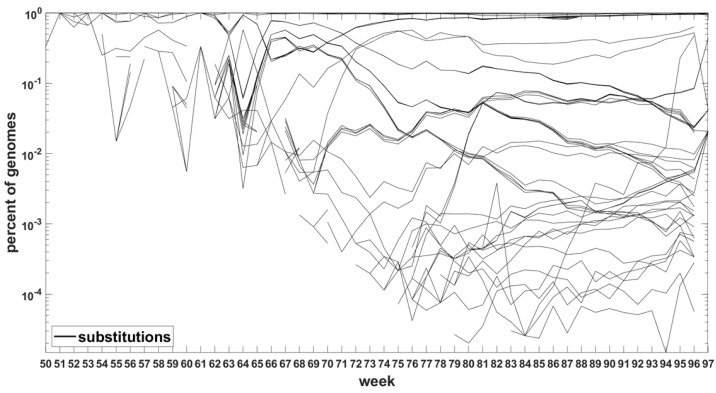
Time trajectories of frequencies of the substitutions among top 1000 mutations observed in Delta genomes. Variant-defining sites excluded.

Similar analysis can be carried out for the Delta VOC genomes as depicted in Figure 8 and Figure A7. The horizontal bands observed in these Figures may belong to subvariants of Delta VOC.

### 3.4. Site Frequency Spectra and Gradual Departure from Neutrality

**Figure 9:**
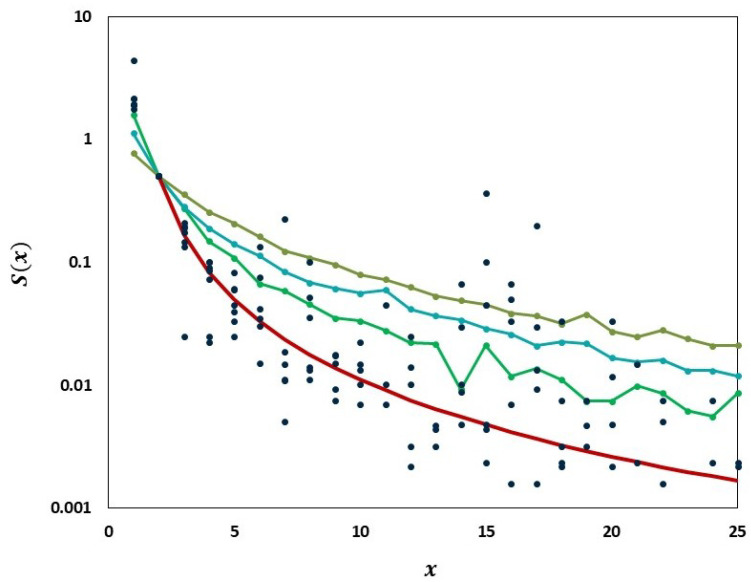
Comparison of the empirical SFS graphs of the Delta VOC genomes at different times to the Griffiths–Tavaré model for small x=1,2,…,25 . Theoretical S(x) computed for the non-singleton part x≥2 . *Red line*, model; *Dark blue dots*, merged SFS points for weeks 64–69; *Green line,* SFS week 74; *Turquoise line,* SFS week 79; *Frosted green line,* SFS week 84.

**Figure 10:**
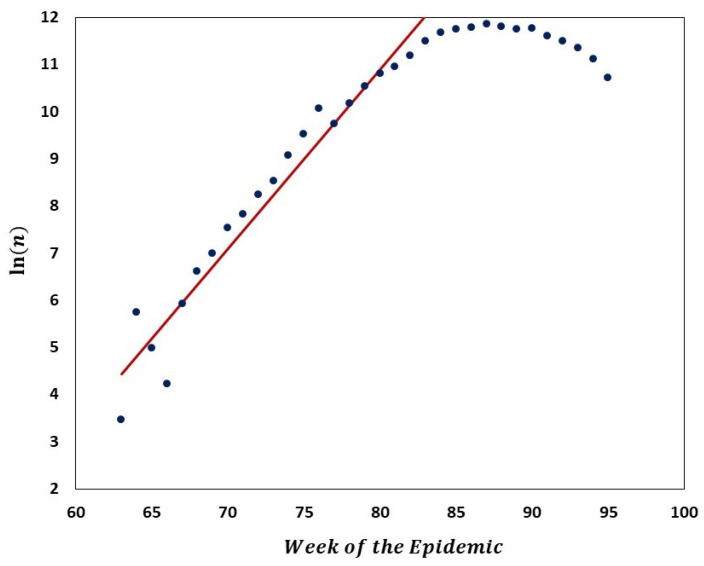
Semi-logarithmic graph of the number of Delta VOC genomes recorded in the interval from 63 to 84 weeks of the pandemic. Red line depicts a linear regression fit to the ascending portion of the curve, indicating approximate exponential growth in that period. Equation of the straight line, ln(x)=0.38×# weeks − 19.5 , which implies ca. 1.82 week genome count doubling time.

We carried out analyses aimed at tracking the gradual departure from neutrality as mutation frequencies are increasing. Figure 9 illustrates increasing departure from the predictions of the Griffiths–Tavaré model (Section 2.4.2) in the course of the evolution of the low-frequency variant sites of the Delta VOC. In the time interval from week 63 to 84, the number of genomes recorded per week varied approximately exponentially, as indicated by the straight-line fit in the semi-logarithmic coordinates depicted in Figure 10. However, while in the earlier weeks the SFS followed the Griffiths–Tavaré model, starting in week 74, the departure from the model became significant and increasing. Let us remember that even in the early Delta VOC history, the fit is limited to the lowest mutation frequencies.

**Figure 11:**
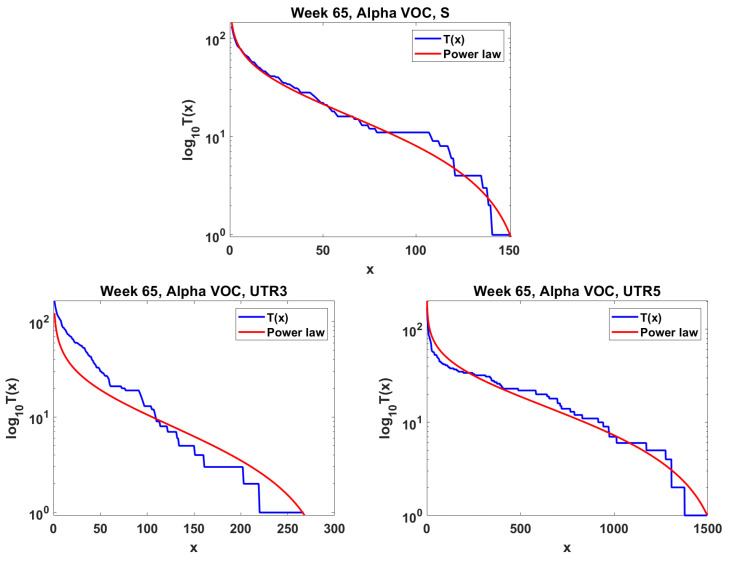
Mid-frequency range SFS tails (blue) in semi-log scale fitted by power-law curves (red). Alpha VOC, week 65. **Top** Spike gene. **Bottom left** Untranslated region 3 ${}^{\prime }$ . **Bottom right** Untranslated region 5 ${}^{\prime }$ .

**Figure 12:**
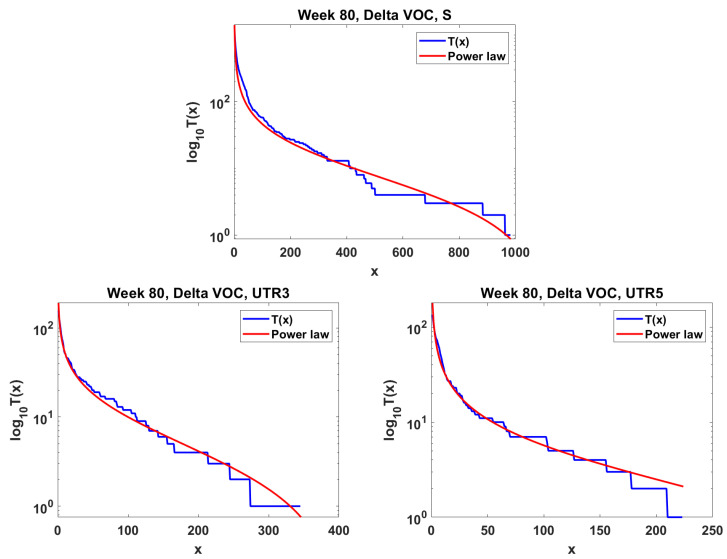
Mid-frequency range SFS tails (blue) in semi-log scale fitted by power-law curves (red). Delta VOC, week 80. **Top** Spike gene. **Bottom left** Untranslated region 3 ${}^{\prime }$ . **Bottom right** Untranslated region 5 ${}^{\prime }$ .

**Figure A6:**
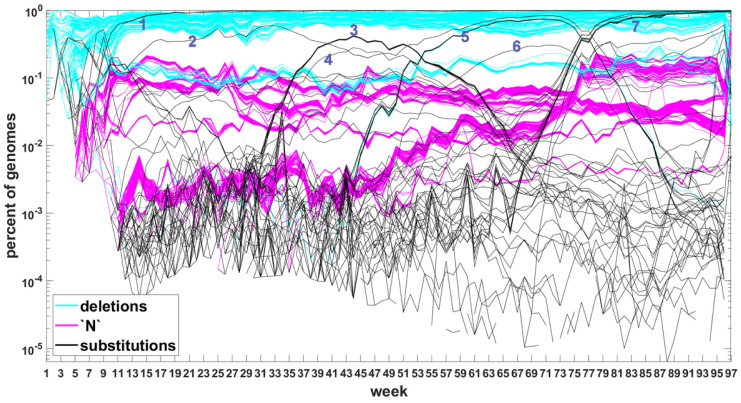
Time trajectories of frequencies of the top 1000 mutation sites of different categories: substitutions (black), deletions (blue), and unknown (magenta) observed in all genomes. Variant-defining sites excluded. Numbers next to the characteristic bands correspond to the “Band id” column in Table A7 and Table A8.

The next step is the extension of the analysis beyond the lowest frequencies, with exclusion of mutations characterizing the macroevolution of the VOC that are characterized by the ∩-shaped “bands” in Figure 7, Figure A5 and Figure A6, discussed earlier on. We discuss the results for the Alpha (Figure 11) and Delta (Figure 12) VOC here. In both cases, the SFS tails are approximated by the power-law curves discussed in the Methods section, which indicate selection replacing more viable variants with more indolent variants, constant α>1 . The strength of selection varies between Alpha and Delta VOC, with corresponding α≈1.2 and α≈1.6 . The Beta VOC’s SFS seem too rugged to provide meaningful estimates.

**Figure 13:**
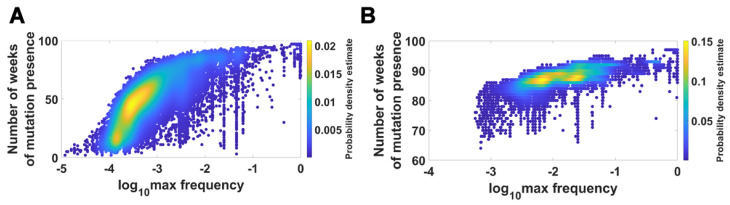
Relationship between maximum frequency of mutations and the number of weeks for which mutations were recorded. *All genomes.* (**A**) mutations and deletions; (**B**) mutations, deletions and non-identified (N).

We visualized the relationship between maximum frequency of mutations (including deletions) and the number of weeks over which the mutation was recorded. It is depicted by the color-coded isoclines in Figure 13 for all genomes (for VOC genomes see the Appendix B Figure A8). The shape of the yellow “ridges” in the figure indicates a positive correlation. This is a manifestation of an intuitively clear rule: Mutations that are more frequent persist for a shorter time. The details of why and how this happens are less clear. We return to the subject in the Discussion.

**Figure 14:**
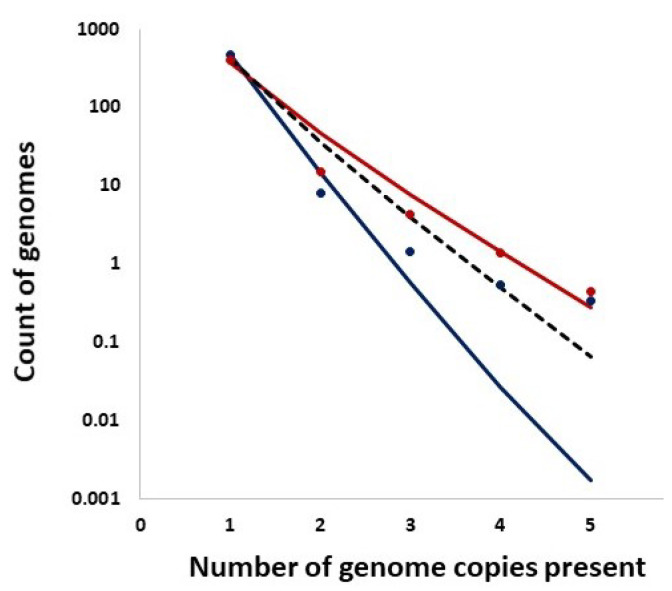
Distributions of the counts of entire genomes present in a given number of copies (singletons, doublets, etc.). *Solid Lines:* Ewens sampling formula under Infinite Allele Model (IAM) in mutation–drift equilibrium. *Bullets*: Data. *Color coding:* Red: week 69, Navy: week 75. *Dashed Line:* Griffiths–Pakes under IAM and exponential growth.

In addition, we studied distributions of the counts of entire genomes present in a given number of copies and the expected counts from the Ewens sampling formula under IAM in mutation–drift equilibrium as well as from the Griffiths–Pakes under IAM and exponential growth (see Section 2 and Figure 14). Remarkably, both methods provide singleton counts similar to those recorded, while the further distribution terms vary.

### 3.5. Tug-of-War Model

In this section, we address the question of if we can reproduce, at least qualitatively, the patterns of genome variant rise and decline represented by the ∩-shaped patterns in Figure 7 and Figure A6 by a simple model based on principles of population genetics. Let us notice that we are not modeling the spread of epidemic expressed as the number of infected cases but the spread of genome variants.

The modeling purpose is to show how new variants (clones) stem from rare advantageous driver mutations, and then acquire neutral or disadvantageous passenger mutations, which gradually reduce the fitness of the variant, which can be then outcompeted by a new variant due to other driver mutations. We adopt the Tug-of-War model of McFarland et al. [24] in the Moran process version, called Model B in Kurpas and Kimmel [34], where it was used to understand clone succession in cancer. The mathematical framework is laid out in Section 2.6 in Section 2.

In summary, the parameters of the models are as follows:

- *N* population size (number of genomes);
-
μ mutation rate per genome;
- *p* probability that mutation is an advantageous driver, 1−p probability that mutation is a deleterious passenger;
- *s* the selective advantage of the driver, and *d* the selective disadvantage of the passenger.

**Figure 15:**
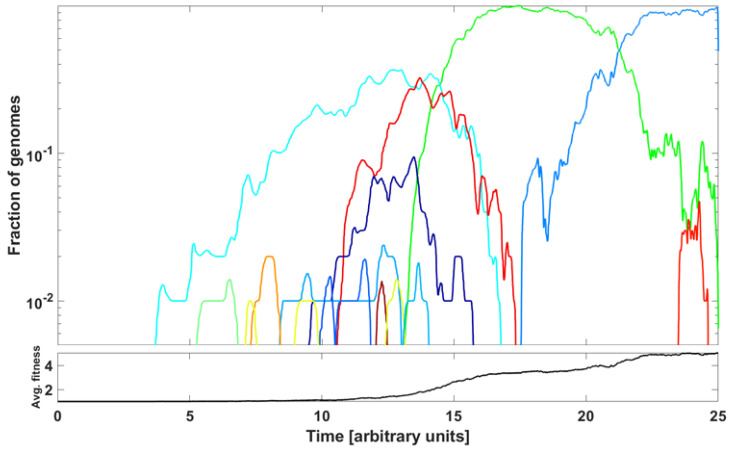
Frequencies of clones initiated by driver mutations in McFarland’s Tug-of-War stochastic process (Model B from Kurpas and Kimmel 2022 [34]) modeling evolution of a population of *N* genomes. The graph depicts relative frequencies, in semi-logarithmic scale, of genome clones depicted in different colors. Rules of the process: Relatively rare advantageous driver mutations (rate pμ per genome, with *p* small) increase genome fitness by factor 1+s , s>0 , while more frequent neutral or deleterious passenger mutations (rate (1−p)μ per genome) decrease genome fitness by factor 1−d , d≥0 . At rate λ=1 per genome, a genome dies and becomes replaced by another genome, randomly chosen with probability proportional to replacement’s fitness. Coefficient values: N=100 , μ=0.1 , p=0.1 , s=0.5 , d=0.0 and time in arbitrary units. We observe a succession of clones outcompeting each other, with the average fitness of genomes in the population increasing with time.

An example of outcome is illustrated in Figure 15. The parameter values are listed in the caption to the Figure. We observe a succession of emerging and receding driver-initiated clones (variants) of genomes, as the average fitness of the population increases with time. Visually, it resembles the train of the ∩-shaped patterns in Figure 7 and Figure A6. The significance of the modeling is discussed in the next section.

## 4. Discussion

In this study, we accumulated and aligned 4.7 million SARS-CoV-2 genomes from the GISAID database and carried out a comprehensive set of analyses. This collection covers the period until the end of October 2021, i.e., the beginnings of the Omicron variant. First, we explored combinatorial complexity of the genomic variants emerging and their timing, indicating very strong, albeit hidden, selection forces. To this end, we analyzed SARS-CoV-2 genomes to determine how individual mutations that define the Alpha, Beta and Delta variants were appearing over time and how these were interfering with neutral and mildly deleterious mutations in different ranges of mutation frequency. Our analyses showed that the VOC-defining mutations did not arise gradually but rather co-evolved rapidly, leading to the emergence of the full VOC strain (Figure 3). We did not observe transient states, which would be expected under neutral evolution. In addition, the recorded assortment of haplotypes involving the VOC-defining mutations demonstrated that maybe around 1% of combinatorially feasible variants appeared in the known viral strains (Table A4, Table A5 and Table A6). These results seem to indicate that segregating sites in the Alpha, Beta and Delta variants evolved under strong positive selection, with a possible contribution of recombinations among viruses carrying subsets of VOC-defining mutations. Research has shown that the latter is common in bat coronaviruses [44] and might indeed also be affecting the evolution of SARS-CoV-2 [45]. Observed mutation patterns may also be due to mutation hotspots, which were detected in the region encoding the Spike protein [46].

As noted in Neher [5], recently, Hill et al. [47] and Tay et al. [48] investigated the dichotomous pattern of SARS-CoV-2 evolution with step-wise evolution within clades or variants and atypical bursts of evolution leading to new variants and showed that the rate of evolution along branches giving rise to new variants is up to four-fold higher than the background rate. However, this does not seem to exclude selection as the underlying mechanism; please see further on.

In addition, we cannot rule out the possibility that genomes carrying subsets of VOC-defining mutations avoided collection and sequencing. In the data gathered by GISAID, we can clearly see temporal differences in the number of sequenced genomes (as shown in Figure A1A), but more importantly, most of the collected genomes come from Europe and the United States. The under-representation of sequences from other parts of the world might alter our conclusions.

**Figure A7:**
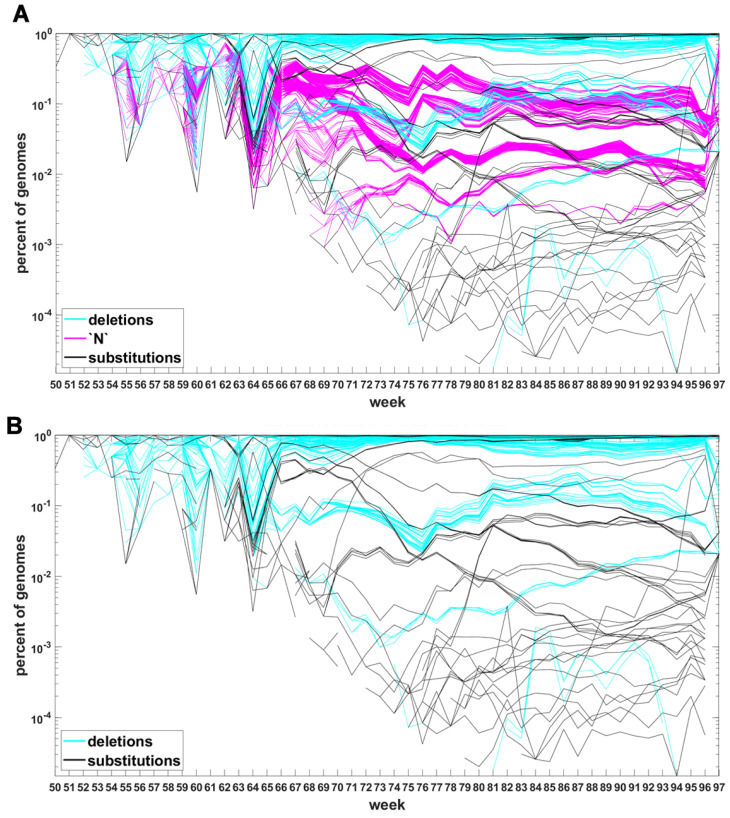
Time trajectories of frequencies of the alterations of specified types among the top 1000 mutations observed in Delta genomes. (**A**) Substitutions (black), deletions (blue) and unknown (magenta); (**B**) substitutions (black), deletions (blue). Variant-defining sites excluded.

**Figure A8:**
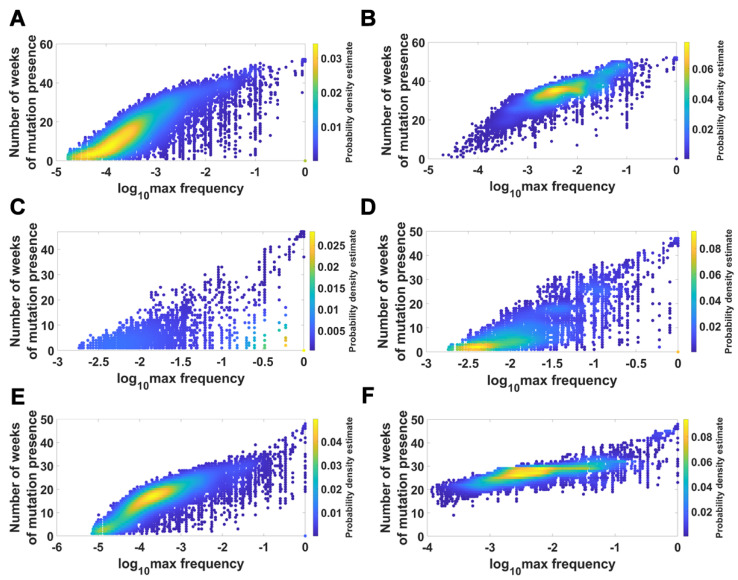
Relationship between maximum frequency of mutations and the number of weeks for which mutations were recorded. *Alpha VOC genomes.* (**A**) mutations and deletions; (**B**) mutations, deletions and non-identified (N). *Beta VOC genomes.* (**C**) mutations and deletions; (**D**) mutations, deletions and non-identified (N). *Delta VOC genomes.* (**E**) mutations and deletions; (**F**) mutations, deletions and non-identified (N).

To explore in some detail the evolutionary forces at work, we developed time trajectories of mutations at all 29,903 sites of the SARS-CoV-2 genome, week by week, and stratified them into trends related to (i) point substitutions, (ii) deletions and (iii) non-sequenceable regions (Figure 7, Figure 8 and Figure A5, Figure A6 and Figure A7). Among others, as mentioned earlier on, this allowed us to track the non-standard variant-defining mutations, left out in the original definitions of the variants of concern.

We focused on classifying the genetic forces active at different ranges of the mutational spectrum. A “reasonable” presumption might be that at the lower end of the mutational spectrum, there exists a “neutral foam” that is affected by mutation and drift, counteracting each other and creating a barrier, prohibiting the evolutionary process from dying out (see further on). Moving further up the frequency spectrum, one might expect forces related to competition and selection show their presence, with negative selection increasing with the size of the VOC genome population and accumulation of deleterious mutations.

As evident from Figure 9, we observe the agreement of the lowest-frequency mutation SFS with the Griffiths–Tavaré theory [22] under the Infinite Sites Model (ISM) and neutrality. This is consistent with the results of IAM testing; the numbers of single-copy haplotypes agree with two models under neutrality, though further terms diverge (Figure 14). If we widen the frequency range, we observe the SFS to be much more consistent with the Tung–Durrett model (Figure 11 and Figure 12), assuming clone competition and selection [23]. The coefficients of the fitting model indicate the possibility of selection acting to promote the gradual growth slowdown, as observed in the history of the VOC.

These results add up to a model of genomic evolution, which partly fits into the classical drift barrier ideas. Classically, drift barrier prevents the mutations from dominating fitness change too easily, as explained in a body of theoretical work in the field of evolutionary genetics, such as [49,50,51]. These papers concern the interplay among mutation, drift and selection, in the absence of recombination (asexual reproduction), where epistasis plays a major role. In our case, a somewhat different barrier, arguably present at the bottom of the mutation frequency spectrum, contributes to injecting mutants, which becomes successful, but then their growth rate decays and they are replaced by others. Certain observations, such as mutations “bands” persistent over the epidemic history, suggest the contribution of genetic forces different from mutation, drift and selection, including recombinations and other genome transformations.

As already mentioned, Neher [5] reviewed the mechanisms of new strain formation in influenza A and HIV-1 viruses and emphasized the exceptional nature of the dichotomous pattern of SARS-CoV-2 evolution with step-wise evolution within clades or variants and atypical bursts of evolution leading to new VOC [47,48]. Furthermore, [5] concluded that a difference in evolutionary rate is only seen for non-synonymous changes, while the rate of synonymous evolution within variants was compatible with that seen between variants. The paper also systematized the knowledge regarding substitution types, leading to new adaptations. These conclusions do not contradict our finding of neutrality at the lowest frequencies of the SFS and gradually picking up negative selection at the mid-range frequencies, as documented in Figure 9, Figure 10, Figure 11 and Figure 12. To synthesize our findings and contribute to the discussion regarding mechanisms of adaptation leading to wave-form succession of the VOC, we proposed a Tug-of-War-type model (see [34] and Section 2.6 for details) in which new variants (clones) stem from rare advantageous driver mutations, and then acquire neutral or disadvantageous passenger mutations which gradually reduce the fitness of the variant, which can be then outcompeted by a new variant due to other driver mutations. Although the current version is a “toy” model, and lacks the resolution necessary for predictive power, it reproduces the succession of clones resembling the Alpha, Beta and Delta pattern (Figure 15) and provides a mathematically consistent mechanism of VOC emergence and replacement.

## Figures and Tables

**Figure A3:**
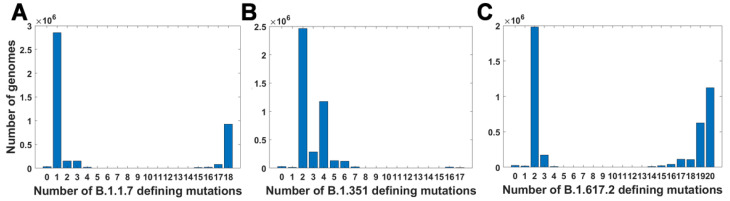
Frequency of genomes carrying a given number of VOC-defining mutations. (**A**) B.1.1.7 (Alpha) variant; (**B**) B.1.351 (Beta) variant; (**C**) B.1.617.2 (Delta) variant.

## Data Availability

We shared tables with week-by-week mutation frequency at given sites (only substitutions; deletions and substitutions) for (1) all genomes, (2) Alpha, (3) Beta and (4) Delta variant. The remaining original contributions presented in the study are included in the article/Appendix, further inquiries can be directed to the corresponding author. Raw data used in the analyses were downloaded from the GISAID database (www.gisaid.org accessed on 30 October 2021).

## Abbreviations

The following abbreviations are used in this manuscript:

SARS-CoV-2	Severe Acute Respiratory Syndrome Coronavirus 2
VOC	Variants of Concern
MSA	Multiple Sequence Alignment
SFS	Site Frequency Spectrum
ISM	Infinite Sites Model
IAM	Infinite Allele Model
ESF	Ewens Sampling Formula

## Appendix A. Figures and Tables

Figure A1 clarifies the relationship among the weekly count of genomes sequenced and succession of the VOC considered in the paper.

Table A1, Table A2 and Table A3 complement Section 1 in the main body of the text.

The remaining portions of the Appendix are organized as a twin reflection (with matching headings) of the sections in the main body of the paper.

**Table A1:** Defining mutations of the B.1.1.7 (Alpha) variant.

Gene	Nucleotide	Amino Acid
ORF1ab	C3267T	T1001I
	C5388A	A1708D
	T6954C	I2230T
	Δ 11288–11296	SGF 3675–3677 deletion
S	Δ 21765–21770	HV 69–70 deletion
	Δ 21991–21993	Y144 deletion
	A23063T	N501Y
	C23271A	A570D
	A23403G	D614G
	C23604A	P681H
	C23709T	T716I
	T24506G	S982A
	G24914C	D1118H
ORF8	C27972T	Q27stop
	G28048T	R52I
	A28111G	Y73C
N	28280–28282 GAT→CTA	D3L
	C28977T	S235F

**Table A2:** Defining mutations of the B.1.351 (Beta) variant.

Gene	Nucleotide	Amino Acid
ORF1ab	C1059T	T85I
	G5230T	K837N
	A10323G	K90R
	Δ 11288–11296	SGF 3675–3677 deletion
	C14408T	P314L
S	C21614T	L18F
	A21801C	D80A
	A22206G	D215G
	Δ 22281–22289	LAL 242–244 deletion
	G22813T	K417N
	G23012A	E484K
	A23063T	N501Y
	A23403G	D614G
	C23664T	A701V
ORF3a	G25563T	Q57H
E	C26456T	P71L
N	C28887T	T205I

**Table A3:** Defining mutations of the B.1.617.2 (Delta) variant.

Gene	Nucleotide	Amino Acid
ORF1b	C14408T	P314L
	G15451A	G662S
	C16466T	P1000L
S	C21618G	T19R
	G21987A	G142D
	Δ 22029–22034	E156–F157 deletion
	A22034G	R158G
	T22917G	L452R
	C22995A	T478K
	A23403G	D614G
	C23604G	P681R
	G24410A	D950N
ORF3a	C25469T	S26L
M	T26767C	I82T
ORF7a	T27638C	V82A
	C27752T	T120I
ORF8	Δ 28248–28253	D119–F120 deletion
N	A28461G	D63G
	G28881T	R203M
	G29402T	D377Y

## Appendix B. Results

### Appendix B.1. VOC Timelines

Figure A2 complements Figure 3 in the main body of the text.

### Appendix B.2. Mutation Subsets and Their Frequency

Table A4, Table A5 and Table A6 and Figure A3 and Figure A4 complement Figure 4, Figure 5 and Figure 6 in the main body of the text.

**Table A4:** Number of unique subsets of VOC Alpha-defining mutations for a given mutation count, out of a total of 18.

Number of Mutations	Expected	Observed
1	18	17
2	153	48
3	816	149
4	3060	299
5	8568	444
6	18,564	447
7	31,824	410
8	43,758	376
9	48,620	409
10	43,758	455
11	31,824	443
12	18,564	511
13	8568	585
14	3060	654
15	816	511
16	153	152
17	18	18
18	1	1

**Table A5:** Number of unique subsets of VOC Beta-defining mutations for a given mutation count, out of a total of 17.

Number of Mutations	Expected	Observed
1	17	15
2	136	49
3	680	82
4	2380	179
5	6188	222
6	12,376	254
7	19,448	182
8	24,310	158
9	24,310	133
10	19,448	128
11	12,376	148
12	6188	182
13	2380	178
14	680	155
15	136	83
16	17	17
17	1	1

**Table A6:** Number of unique subsets of VOC Delta-defining mutations for a given mutation count, out of a total of 20.

Number of Mutations	Expected	Observed
1	20	17
2	190	53
3	1140	85
4	4845	182
5	15,504	272
6	38,760	304
7	77,520	296
8	125,970	313
9	167,960	306
10	184,756	342
11	167,960	384
12	125,970	490
13	77,520	512
14	38,760	580
15	15,504	563
16	4845	510
17	1140	419
18	190	154
19	20	19
20	1	1

### Appendix B.3. Longitudinal Analysis of Mutations in the SARS-CoV-2 Genomes

Figure A5 and Figure A6 complement Figure 7 and Figure A7 complements Figure 8 in the main body of the text.

### Appendix B.4. Site Frequency Spectra and Gradual Departure from Neutrality

Figure A8 complements Figure 13 in the main body of the text.

## Appendix C. Relevant Accession Numbers

NC_045512.2—First genome collected in Wuhan (reference sequence).

EPI_ISL_601443—One of the first Alpha variant genomes collected and submitted to GISAID during the wave in United Kingdom.

EPI_ISL_712073—One of the first Beta variant genome collected and submitted to GISAID during the wave in South Africa.

EPI_ISL_3148365—One of the characteristic Delta variant genomes collected.

## Appendix D. Bands Identified in Longitudinal Plots

**Table A7:** Bands identified in longitudinal plots (all genomes)—part 1.

Band id	Nucleotide	Genome Region	Type of Mutation	Reference
1	C241T	5’UTR	Europe/US spring 2020 wave	[52,53,54,55]
	C3037T	ORF1ab	Europe/US spring 2020 wave	[52,53,54,55]
2	G28882A	N	Europe/US spring 2020 wave	[56]
3	T445C	ORF1ab	synonymous European B.1.177 lineage (2020)	[57]
	C6286T	ORF1ab	synonymous European B.1.177 lineage (2020)	[57]
	G21255C	ORF1ab	synonymous European B.1.177 lineage (2020)	[58,59]
	C22227T	S	European B.1.177 lineage (2020)	[59,60]
	C26801G	M	synonymous European B.1.177 lineage (2020)	[57]
	C28932T	N	European B.1.177 lineage (2020)	[59,60]
	G29645T	ORF10	European B.1.177 lineage (2020)	[59,60]
4	C27944T	ORF8	synonymous mutation enriched in Kappa variant	[61]
5	C913T	ORF1ab	synonymous P.1 (Gamma); synonymous B.1.1.7 (Alpha)	[43]
	C5986T	ORF1ab	synonymous P.1 (Gamma); synonymous B.1.1.7 (Alpha)	[43]
	C14676T	ORF1ab	synonymous P.1 (Gamma); synonymous B.1.1.7 (Alpha)	[43]
	C15279T	ORF1ab	synonymous P.1 (Gamma); synonymous B.1.1.7 (Alpha)	[43]
	T16176C	ORF1ab	synonymous P.1 (Gamma); synonymous B.1.1.7 (Alpha)	[43]
6	A28095T	ORF8	Alpha variant accompanying mutation (possibly sublineage)	[62,63]

**Table A8:** Bands identified in longitudinal plots (all genomes)—part 2. The Table complements Figure A6.

Band id	Nucleotide	Genome Region	Type of Mutation	Reference
7	G210T	5’UTR	Delta extragenic mutation	[64,65]
	G4181T	ORF1ab	non-synonymous mutation; possibly Delta subvariant	[64,65]
	C6402T	ORF1ab	B.1.351.3 (Beta) VOC subvariant; possibly Delta subvariant	[65]
	C7124T	ORF1ab	C.37 (Lambda) VOC; possibly Delta subvariant	[65]
	C8986T	ORF1ab	synonymous mutation; possibly Delta subvariant	[64,65]
	G9053T	ORF1ab	non-synonymous mutation; possibly Delta subvariant	[64,65]
	C10029T	ORF1ab	C.37 (Lambda) VOC; Omicron VOC; possibly Delta subvariant	[64,65]
	A11201G	ORF1ab	B.1.617.1 (Kappa) VOC; possibly Delta subvariant	[64,65]
	A11332G	ORF1ab	synonymous mutation; possibly Delta subvariant	[64,65]
	C19220T	ORF1ab	non-synonymous mutation; possibly Delta subvariant	[64,65]
	C27874T	ORF7b	non-synonymous mutation; possibly Delta subvariant	[64,65,66]
	G28916T	N	non-synonymous mutation; possibly Delta subvariant	[64,65,67]
	G29742T	3’UTR	recombination hotspot	[68,69]
