# Correction: Kurpas et al. Genomic Analysis of SARS-CoV-2 Alpha, Beta and Delta Variants of Concern Uncovers Signatures of Neutral and Non-Neutral Evolution. *Viruses* 2022, *14*, 2375

**DOI:** 10.3390/v15051047

**Published:** 2023-04-25

**Authors:** Monika Klara Kurpas, Roman Jaksik, Pawel Kuś, Marek Kimmel

**Affiliations:** 1 Faculty of Automatic Control, Electronics and Computer Science, Silesian University of Technology, Akademicka 16, 44-100 Gliwice, Poland; 2 Department of Statistics and Bioengineering, Rice University, 6100 Main Street, Houston, TX 77005, USA

**Missing Funding**

In the original publication [1], the funder Polish National Science Center, 2021/41/B/NZ2/04134 to Marek Kimmel (MK) was not included. The correct funding should be as follows: 

“This research was funded by a subsidy for the maintenance and development of research potential BKM-538/RAU1/2022 (02/040/BKM22/1031) granted by the Polish Ministry of Science and Higher Education (M.K.K), the Polish National Science Center grant 2018/29/B/ST7/02550 (R.J.), the European Union through the European Social Fund grant POWR.03.02.00-00-I029 (P.K.), the NSF/DMS Rapid Collaborative grant NSF/DMS-2030577 to Marek Kimmel and Simon Tavaré (M.K.), and the Polish National Science Center grant 2021/41/B/NZ2/04134 to Marek Kimmel (M.K.).”

**Text Correction**

There was an error in the original publication. Practical computations of the SFS based on Tung–Durrett model were carried out using notation $\alpha =1+\lambda_{0}/\lambda_{1}$. However, this is inconsistent with the original notation $\alpha =\lambda_{0}/\lambda_{1}$. The correction makes expressions (6) and (7) consistent with the original notation.

A correction has been made to Materials and Methods, SFS under Birth-and-Death Process Model with Mutation and Selection, paragraph 1:

Under these hypotheses, if the fitnesses of the two types are λ_0_ < λ_1_, then the site frequency spectrum approximately follows the power curve, in our notation

(6) $$S\left(x\right)={Cx}^{-(1+\alpha )},x=1,\ldots ,n$$

hence the tail *T*(*x*) = *∑*_*ξ*>*x*_ *S*(*ξ*) follows the law

(7) $$T\left(x\right)=\frac{C}{\alpha }\left(n^{-\alpha }-x^{-\alpha }\right),x=1,\ldots ,n$$

where *α* = *λ*_0_/*λ*_1_.

The authors state that the scientific conclusions are unaffected. These corrections were approved by the Academic Editor. The original publication has also been updated.
